# Research training in child and adolescent psychiatry: lack of motivation or a structural problem?

**DOI:** 10.1007/s00787-023-02293-7

**Published:** 2023-09-22

**Authors:** Jochen Kindler, Michael Kaess, Stephan Eliez, Maya Cosentino, Matthias Liebrand, Paul Klauser

**Affiliations:** 1 University Hospital of Child and Adolescent Psychiatry and Psychotherapy, University of Bern, Bern, Switzerland; 2 Department of Child and Adolescent Psychiatry, Centre for Psychosocial Medicine, University Hospital Heidelberg, Heidelberg, Germany; 3 Department of Psychiatry, Service of Child and Adolescent Psychiatry, Lausanne University Hospital and the University of Lausanne, Lausanne, Switzerland; 4 Department of Psychiatry, Center for Psychiatric Neuroscience, Lausanne University Hospital and the University of Lausanne, Lausanne, Switzerland; 5 Department of Psychiatry & Behavioral Sciences, Stanford University, Stanford, CA USA; 6 Fondation Pôle Autisme, Département de psychiatrie, Faculté de Médecine, Université de Genève, Genève, Switzerland

Research is essential for the continuous improvement of our current practices in child and adolescent psychiatry (CAP)—it is the basis for more accurate early detection and diagnosis of mental disorders as well the refinement and tailoring of our preventive and therapeutic strategies.

The current issue of European Child and Adolescent Psychiatry demonstrates how research can impact clinical practice. For example, the case–control study by Wan-Chen Lee et al. investigated healthcare contacts and comorbid disorders in 159 adolescent suicides and nearly 3200 matched controls [1]. Patient health care utilization was significantly higher during the 3 months prior to suicides and higher rates of mental but also physical comorbidities were observed among those who committed suicide than among controls. Thus, patients seeking help could potentially be identified as at risk and benefit from suicide prevention measures. The work of Marie Deserno et al. [2] challenges the traditional distinction between autism spectrum disorders and attention deficit/hyperactivity disorder using a novel, data-driven approach to describe and understand psychiatric nosology. Suzanne de Jong [3] investigated the effectiveness of a new, self-help parenting program that integrates psychoeducative and behavioral parenting techniques for children with externalizing behaviors. Fifteen weeks after program completion, significantly higher effects that lasted at least 7 months were found in the intervention group compared to the control group. These research findings are just three examples from the collection of highly relevant publications in this issue that demonstrate that research has the potential to be translated into clinical practice in meaningful ways.

While research generally arouses interest and enthusiasm among doctors and can be a stimulating factor that enriches the work environment, research can also be perceived as laborious and cumbersome [4]. Despite the fact that research training is one of the four consensus priorities defined by the World Psychiatric Association, the World Association for Infant Mental Health, the United Nations Special Rapporteur on the Right to Health (WHO) and Child and Adolescent Psychiatry (CAP) international societies, CAP’s research culture is relatively underdeveloped in comparison to other medical specialities [5]. Is this situation related to resident’s attitudes towards and interests in research or the way they perceive their career opportunities? What are the parameters facilitating or hindering research from their point of view?

To shed light on these questions, a survey was conducted in September 2022 at the Residents’ Day of the Swiss Society of Child and Adolescent Psychiatry and Psychotherapy Congress in Zürich. CAP residents from all regions of Switzerland attended the Residents’ Day and answered questions about their involvement in research and factors that could influence it. The survey aimed to evaluate current and past research activities, subjective attitudes towards research as well as basic demographic information. All the participants were enrolled in a CAP residency program somewhere in Switzerland and a total of 97 CAP residents completed the survey.

About two-thirds of the participants had received their medical degree outside of Switzerland (62.9%). Their place of work was in 1 of 16 Swiss cantons, and their primary language was Italian, French, or German. The largest percentage worked in cantons with a university hospital (i.e., Geneva, Vaud, Zurich, Bern, and Basel). About a third of the participants were in their first year of training (30.9%), a minority (9.3%) was nearing its completion (i.e., 6th year or later), and about 60% were equally distributed between their second and fifth year of training.

About a quarter of the residents had completed a doctoral thesis (27.8%, MD *n* = 26, MD-PhD *n* = 1) but most of those holding a doctoral degree were not currently involved in research activities (74.1%). Among residents without a doctoral degree (*n* = 70), 25.7% were currently registered as an MD or PhD candidate. Among those who were not registered, 42.1% were planning to do a thesis at some point in their career and 35.1% did not know. Only a minority (22.8%) did not intend to write a thesis, the main reasons being “not seeing the point of completing a thesis” (38.5%), “not knowing anyone who could supervise a thesis" (30.8%), and lack of time (23.1%). Although almost half of the participants (44.3%) were aware of colleagues doing research, less than a quarter of them (22.7%) previously participated in research at their current workplace.

Among those not involved in research (*n* = 75), only 13.3% could name and briefly describe at least one project currently underway at their workplace, despite that almost two-thirds (64%) indicated that research was of importance at their institution. Only 8 (10.7%) of the participants not involved in research indicated that research (e.g., clinical studies, meta-analyses, or reviews) was regularly discussed, while 17 (22.7%) indicated that research was never discussed or shared by their supervisors at work.

In contrast, most residents involved in research could name and briefly describe at least one research project (59.1%) and indicated that research is valued and promoted at their institution (90.9%). However, among those involved in research, only 18.2% reported that scientific results were discussed regularly and reported that research was never discussed by their supervisors.

Among the residents involved in research (*n* = 22), the majority help recruit patients for research projects (63.6%), collect data (54%), analyze data (54%), and write manuscripts (54%). Time per week dedicated to research among the residents was reported to be very minimal (i.e., < 1 h) for nearly half (45.5%), half a day for a about a fifth (18.2%), 1 day for about a quarter (22.7%) and 2 days or more for a minority (13.6%, see Fig. 1).

**Fig. 1:**
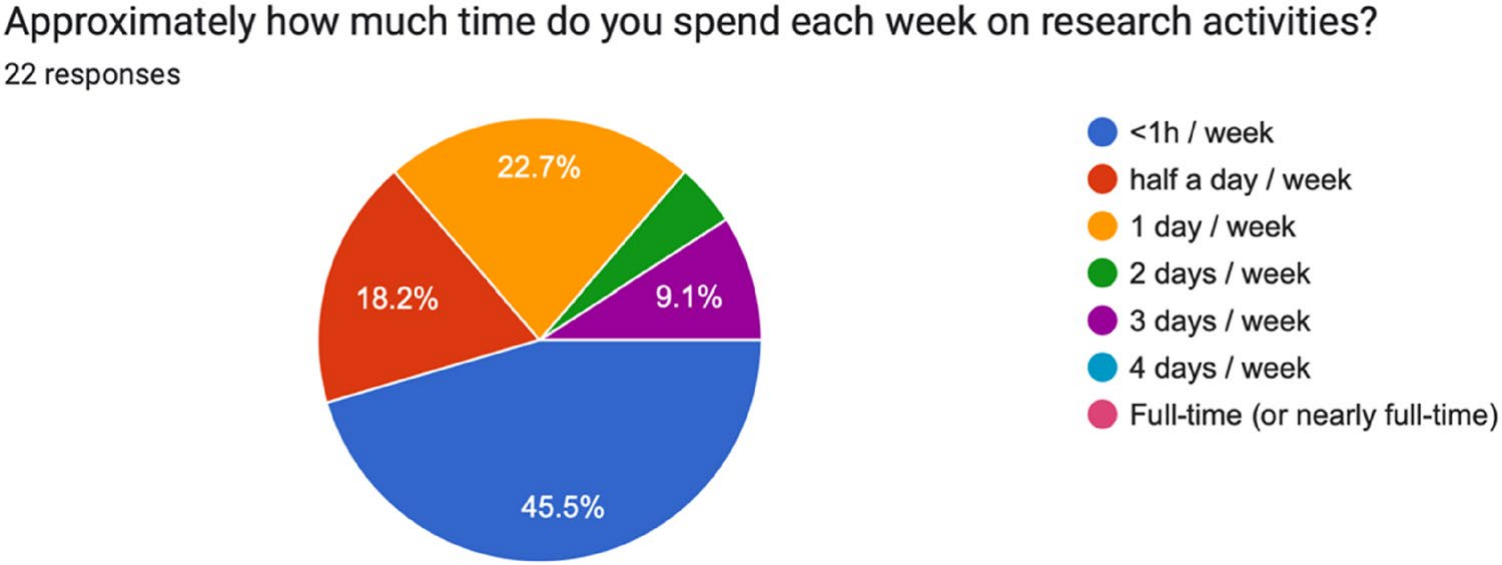
Week time dedicated to residents’ research

Overall, these outcomes have several implications:

- The vast majority of CAP residents plan to participate in research. Only a minority of those not already holding a doctorate or being registered for a thesis have no intention to write a thesis at some point in their career.
- Apart from the lack of motivation, the main reasons residents do not plan to participate in research are related to lack of time and not knowing someone who could supervise their work.
- Most residents who are involved in research believe research is important and research activities and/or the discussion of research would have a positive impact (e.g., on their clinical work).
- However, research support and research teaching norms do not exist in many working environments (i.e., supervisors do not commonly discuss research questions or act as thesis supervisors).

Thus, we conclude there is an untapped potential in the current generation of CAP residents. A substantial number of residents are interested either in personally engaging in research activities and/or discussing research findings. However, there seems to be unmet needs of CAP residents that should be addressed, such as the availability of research supervisors and teachers.

Importantly, our survey did not cover several factors known to influence resident’s willingness to get involved in research. In a recent study conducted in Australia [6], barriers to research for residents were identified as having poor methodological and statistical skills and limited research resources. Health professionals considered financial and organizational support as well as clearly defined research time highly important. Further, the acquisition of research competences and the feeling of autonomy, e.g., having control over own’s personal goals, were relevant. The authors also investigated the motivation to do research. While previous generations might have been motivated by extrinsic factors such as career progression and academic improvement, the highest research-oriented group of the current generation was motivated by future usefulness of their work, attainment (i.e., doing well) and personal enjoyment [7]. A further crucial factor promoting research activities was a feeling of connectedness to other researchers/groups or mentors in a collaborative sense. The most highly motivated group was genuinely curious, willing to produce and share new knowledge, interested in improving patient health outcomes and satisfied with their jobs.

One important question is to define the best time to initiate the support of a person intending to pursue a scientific career. Here, data consistently recommend starting as early as possible, e.g., during medical studies [8]. Most physicians who pursue careers in research have already decided to do research before the start of their residency (e.g., they had a mentor while they were studying, worked in research > 5 h/week and developed their own projects [9]. Studies indicate that there may be a relationship between perceptions of and motivation to do research, e.g., in an environment where research is appreciated, the motivation to do research is high.

Finally, sex inequality in research continues to be a reality. In academic environments, women consistently have lower grant award rates and larger teaching, administrative, and organizational workloads compared to men, in addition to domestic responsibilities (e.g., child care) known to hinder women from pursuing scientific careers. Therefore, special attention is to be directed towards alleviating impediments specific to women [10].

If we want to improve the current status quo of research activities of residents in European child and adolescent psychiatry, what are the next steps?

We recommend the heads of child and adolescent psychiatric facilities to advocate for the inclusion of research and discussion of modern scientific literature in CAP daily clinical work and training. Identifying and supporting those residents who would like to participate will also be essential. Role models and mentoring systems for young clinicians interested in developing a scientific career could also be helpful. Finally, providing the personal and technical resources and—maybe more importantly—the time for young residents to develop their own research ideas, to expand their methodological skills and to follow a science-oriented career track will be essential [11].

Thus, there is work to do—let us start now!

## Data Availability

The data that support the findings of this study are available on request from the corresponding author, JK and PK.
